# Financial toxicity among breast cancer survivors receiving care during the pandemic

**DOI:** 10.1007/s00520-026-10819-3

**Published:** 2026-07-06

**Authors:** Chiara Acquati, Tzuan A. Chen, Chelsea Sanchez, Isabel Martinez Leal, Anastasia Rogova, Shahnjayla K. Connors, Matthew P. Banegas, Grace Smith, Lorraine R. Reitzel, Lorna H. McNeill

**Affiliations:** 1 Eli Coleman Institute for Sexual and Gender Health, University of Minnesota Medical School, Minneapolis, MN USA; 2 Graduate College of Social Work, University of Houston, 3511 Cullen Blvd, Houston, TX 77204-4013 USA; 3 Department of Family Medicine and Community Health, University of Minnesota Medical School, Minneapolis, MN USA; 4 Department of Clinical Sciences, Tilman J. Fertitta Family College of Medicine, University of Houston, 5055 Medical Cir, Houston, TX 77204 USA; 5 Department of Health Disparities Research, The University of Texas MD Anderson Cancer Center, 1515 Holcombe Blvd, Houston, TX 77030 USA; 6 Department of Psychological, Health, and Learning Sciences, University of Houston, McElhinney Hall, 3623 Cullen Blvd., Houston, TX 77204 USA; 7 HEALTH Research Institute, University of Houston, Martin Luther King Blvd., Houston, TX 77204 USA; 8 Department of Behavioral Science, The University of Texas MD Anderson Cancer Center, 1155 Pressler Street, Houston, TX 77030 USA; 9 Department of Health Services Research, The University of Texas MD Anderson Cancer Center, Houston, TX USA; 10 Department of Social Sciences, University of Houston-Downtown, One Main Street, Houston, TX 77002 USA; 11 Department of Radiation Medicine and Applied Sciences, University of California San Diego, San Diego, CA USA; 12 Department of Gastrointestinal Radiation Oncology, The University of Texas MD Anderson Cancer Center, Houston, TX USA

**Keywords:** Financial Toxicity, Distress, Breast Cancer, Cancer Care, COVID-19 Pandemic

## Abstract

**Purpose:**

To appraise financial toxicity (FT) among women receiving care for breast cancer during the COVID-19 pandemic and determine sociodemographic, clinical, psychosocial, and care-disruption factors linked to greater financial hardship.

**Methods:**

Forty-eight survivors (mean age = 45.3 ± 10.9 years; 42% Black, 42% non-Hispanic White, 17% Hispanic/Latina) completed an online survey. FT was measured with the COST-FACIT. Additional instruments assessed perceived stress (PSS-10), coping (Brief-COPE), self-efficacy (CBI-B), social isolation (PROMIS-SF-4a), health-related quality of life (FACT-B), and pandemic-related delays or changes in cancer care. Pearson correlations explored bivariate associations; backward-selection regression identified predictors of FT.

**Results:**

Mean COST score was 21.77 ± 12.33 and 58.3% of survivors met the threshold for mild or moderate FT (< 26). Worse FT correlated with higher psychological distress (*p* = 0.001), greater social isolation (*p* = 0.005), and more care disruptions (*p* < 0.001). Financial security correlated with greater self-efficacy (*p* = 0.019) and higher health-related quality of life (*p* < 0.001). In multiple regression analysis (R^2^ = 0.69), survivors reporting worse quality of life, lower income, and less education reported greater financial toxicity.

**Conclusions:**

Breast cancer survivors experienced substantial FT while navigating care during the pandemic, with greater burden associated with psychological distress, social isolation, care disruptions, and lower socioeconomic status. FT was attenuated among those with higher health-related quality of life and self-efficacy. Integrating proactive financial navigation, psychosocial screening, and targeted support for socioeconomically vulnerable survivors into routine oncology care may mitigate financial hardship and improve overall well-being.

## Introduction

Cancer survivorship in the United States is growing rapidly: an estimated 18.1 million people are living with a prior cancer diagnosis today, a number projected to reach 26 million by 2040 as the population grows older, screening improves, and treatments advance [1, 2] With longer survival comes rising costs. National spending on cancer care is expected to exceed $245 billion by 2030, a 34 percent increase since 2015 [3]. In 2018 alone, survivors paid approximately $5.6 billion out-of-pocket for surgery, radiation, systemic therapies, and related services [4]. Against this backdrop, the economic burden of cancer, now widely labeled as financial toxicity (FT), has become a recognized clinical and policy priority [5–12].

FT is a multi-faceted construct referring to material, psychological, and behavioral hardship [4, 10, 12–14]. Across studies, approximately 50% of cancer survivors report experiencing finance-related strain [7–9], which negatively impacts their mental health and overall quality of life [5, 6, 10, 11, 15]. Material hardship extends beyond the direct costs of cancer care (e.g., co-payments for treatment) to also include indirect costs (e.g., transportation, lodging, medical supplies, loss of wages or income, and caregiving services) [4]. Cancer survivors experiencing material hardship report borrowing money, spending less on basic goods or leisure activities, incurring debt, using savings, and filing for bankruptcy to cope with the costs associated with care [4, 7, 9, 15, 16]. Financial strain and psychological morbidity are closely linked. In U.S. adults aged 18–64, cancer survivors were more likely than peers without a cancer history to worry about paying medical and routine care costs (53.5% vs 47.1%; *p* < 0.001) [12]. Among survivors experiencing financial hardship, anxiety and depression were markedly more prevalent than in those without hardship [6]. Furthermore, FT can lead to missed or delayed care, such as postponing preventive services, being unable to fill prescriptions or adhering to medication regimens, missing treatments, tests, or follow-up appointments [7, 9, 11, 15].

Evidence demonstrates that FT is prevalent among young adult and adult populations, as well as individuals with lower socioeconomic status and minoritized racial and ethnic identities [16, 17]. Yabroff et al. [16] found higher rates of material and psychological hardship among cancer survivors aged 18 to 64 years than survivors aged 65 and older. Among those of working age, individuals who reported being younger, female, and non-White as well as having experienced a change in employment due to cancer were more likely to report experiencing material hardship. Moreover, individuals who lacked health insurance, had lower family income, and received recent treatment were more likely to report experiencing psychological hardship [16]. Biddell et al. [17] examined racial and ethnic differences in the financial consequences of cancer-related employment disruption. Income loss was more common among Black and Hispanic/Latinx participants (75% each) than among non-Hispanic White participants (60%). Compared to their White counterparts, Black and Hispanic/Latinx participants were also significantly more likely to experience substantial income loss and changes in health insurance coverage.

Although FT is prevalent among survivors across all forms of cancer, individuals diagnosed with breast cancer are particularly at risk of experiencing adverse financial outcomes [11, 18, 19]. Treatment often entails multimodal regimens, frequent specialist visits, and prolonged surveillance, all of which generate substantial direct and indirect costs [18]. Social norms around caregiving and gendered wage disparities compound the risk, leaving many women with fewer financial reserves [4, 9, 20–22]. Age and race further shape the burden: younger survivors, Black, Asian, or other non-White women consistently report higher FT scores and greater cost-related non-adherence than older or White counterparts [19, 23–25].

The COVID-19 pandemic exacerbated financial stress among cancer survivors [11, 26, 27]. For example, among young adults, 36% reported increased credit debt, 19% reported not having money to pay for medical expenses, losing their job or being furloughed, and 17% experienced food insecurity [11]. Racial and ethnic disparities in FT among cancer survivors were also evident, with Black (18%), Asian American (13%), and mixed race/ethnicity (17%) cancer survivors reporting higher levels of FT than non-Hispanic White (8%) and Hispanic (5%) survivors [26].

Despite growing recognition of cancer survivors’ increased risk of experiencing FT during the pandemic, critical knowledge gaps remain regarding the multilevel factors that contribute to this phenomenon among racially and ethnically minoritized breast cancer survivors. Existing research often relies on clinical samples and fails to fully account for the sociocultural and structural conditions that shape survivors’ financial experiences. Moreover, few quantitative contributions have incorporated community-engaged approaches or included racially and ethnically diverse survivors most affected by health inequities. This study addresses these gaps by analyzing quantitative data from a community-based sample of Black, Hispanic/Latina, and non-Hispanic White breast cancer survivors. Guided by the National Institute on Minority Health and Health Disparities (NIMHD) Research Framework [28], the study examines FT through intersecting domains and levels of influence; including individual, interpersonal, and distal factors. By grounding the analysis in a multidimensional framework, this research provides critical insights to inform interventions aimed at reducing financial hardship in breast cancer care.

## Materials and methods

### Procedure

Study materials (flyers, recruitment scripts, and the survey questionnaire) were developed with input from a Community Research Advisory Board and an External Advisory Board, whose feedback shaped wording and outreach strategies (healthrcmi.com/CRAB; uhandpartnership.com/external-advisory-board). This study was reviewed and approved by the Institutional Review Board of the University of Houston (STUDY00002665, approval date 12/15/2020). Recruitment occurred between April 2021 and August 2022 through community-based organizations, breast cancer advocacy groups, social-media posts, church and neighborhood bulletin boards, and two oncology clinics that predominantly serve minority women. Eligible participants were self-identified women who were non-Hispanic Black/African American, Hispanic/Latina, or non-Hispanic White; age ≥ 18 years; diagnosed with stage I–III breast cancer on or after January 2020; currently receiving breast cancer care at enrollment; able to speak and read English; and with access to an internet-enabled device. Women were excluded if they had metastatic (stage IV) disease, cognitive impairment or severe mental illness precluding participation or inability to provide informed consent. Interested women completed an online screening form; those meeting criteria provided informed consent before proceeding with the online survey. Participants were compensated with a $30 Amazon gift card.

### Instruments

#### Financial toxicity

Financial toxicity was measured with the 12-item Comprehensive Score for Financial Toxicity (COST)–FACIT instrument [29]. Items are rated on a five-point Likert scale (0 = not at all to 4 = very much). Total score ranges between 0 and 44, and a higher value implies better financial well-being (i.e., lower FT) (Comprehensive Score for financial Toxicity [COST] scoring Guidelines, Version 2, available online at www.facit.org). For interpretability, we applied the grading system proposed by De Souza et al. [30, 31] and D’Rummo et al. [32]: Grade 0 (≥ 26): Absent or minimal financial impact; Grade 1 (14–25): Mild financial toxicity, indicating some financial hardship; Grade 2 (1–13): Moderate financial toxicity, suggesting significant financial strain; Grade 3 (0 points): Severe financial toxicity, indicating a high degree of financial distress and hardship.

#### Psychological distress

The 10-item Perceived Stress Scale (PSS-10; [33]) was used to measure distress experienced by the respondents. Each item is rated on a five-point scale ranging from 0 = never to 4 = very often. Four positively worded items are reverse-scored and summed with the six negatively worded items to yield a total score between 0 and 40, with higher scores reflecting greater perceived stress. The PSS-10 shows good internal consistency (α ≈ 0.78–0.91) and adequate test–retest reliability (r ≈ 0.55–0.85) [34]. Factor analytic studies consistently replicate a two-factor structure (*Perceived Helplessness* and *Perceived Self-Efficacy*) in diverse cultural contexts [35, 36]. Convergent validity is supported by positive associations with stressful life events, anxiety, and depressive symptoms [34, 37]. The scale has been validated for use across the lifespan and demonstrates measurement invariance across gender, race/ethnicity, and language groups [35, 38].

#### Coping

Coping was measured with the Brief COPE [39], a multidimensional measure including 28 items assessing 14 coping dimensions. Each item is rated on a four-point scale (1 = “I haven’t been doing this at all” to 4 = “I’ve been doing this a lot”). Consistent with the three-factor structure [40, 41] we computed mean scores for *Problem-focused Coping* (active coping, planning, use of instrumental support, positive reframing), *Emotion-focused Coping* (use of emotional support, venting, humor, acceptance, religion, self-blame), and *Avoidant Coping* (self-distraction, denial, substance use, behavioral disengagement). Higher scores on each domain indicate greater reliance on that coping style. The Brief-COPE demonstrates adequate-to-excellent internal consistency for the three composites (α ≈ 0.70–0.90) and has shown convergent validity with distress, quality-of-life, and functional outcomes across diverse stressors, including serious illness [39, 41].

#### Self-Efficacy

The 12-item Cancer Behavior Inventory-Brief Version (CBI-B) assessed respondents’ self-efficacy in managing the illness. Respondents indicate how confident they are that they can carry out specific adaptive behaviors on a nine-point scale (1 = “not at all confident” to 9 = “totally confident”). Item scores are summed to yield a total score ranging from 12 to 108, with higher values denoting stronger perceived capability [42–44]. The scale has excellent internal consistency (α = 0.84–0.88) and adequate test–retest reliability (r ≈ 0.78) [44, 45]. Construct validity is supported by positive correlations with optimism, benefit finding, and quality of life and by negative correlations with depressive symptoms and cancer-related distress [43, 44, 46, 47].

#### Health-related quality of life (HRQOL)

Health-related quality of life was assessed with the Functional Assessment of Cancer Therapy-Breast (FACT-B) Scale, a well-validated 37-item instrument measuring physical, social/family, emotional, and functional well-being, along with breast cancer-specific concerns [48]. Items are rated on a 5-point Likert scale ranging from 0 (“not at all”) to 4 (“very much”), with higher scores indicating better quality of life. Excellent internal consistency was reported for the total score (α ≈ 0.90) and evidence supports construct, convergent, and known-groups validity [48].

#### Social isolation

The four-item PROMIS® Social Isolation Short Form v2.0 (SF-4a) [49] was utilized to assess social isolation. Participants indicated how often they (a) felt left out, (b) believed that people barely knew them, (c) felt isolated from others, and (d) sensed that others were physically present yet not “with” them over the past month. Each item is rated on a five-point Likert scale ranging from 1 = never to 5 = always. Item scores (range = 4–20) were summed and converted to PROMIS T-scores (M = 50, SD = 10); with higher scores representing greater perceived isolation. The scale demonstrates excellent internal consistency (α ≈ 0.90) and strong convergent validity across cohorts with and without chronic illness [49]

#### Cancer care disruptions

Cancer care disruptions were measured with a series of questions investigating the impact of the COVID-19 on access to healthcare services, treatment, and transition to telemedicine. Questions were adapted (with permission) from the ACS CAN COVID-19 Impact on Cancer Patients and Survivors survey.

### Data analysis

Descriptive statistics were calculated to illustrate the sample characteristics in terms of socio-demographic/clinical factors, FT, psychological distress, coping behaviors, self-efficacy, HRQOL, social isolation, and cancer care disruptions. Bivariate correlations of key variables of interest were computed. Internal reliabilities were assessed using Cronbach’s alpha (Cronbach’s α, acceptable > 0.7). Finally, a backward selection linear regression model with a retention criterion for the main effect of *p* < 0.1 was used to investigate which variables best contribute to explaining FT under analysis in the present sample. Three demographic variables included in the model are race, educational level, and income. All analyses were performed using SAS 9.4 and significance level was set at *p* < 0.05.

## Results

### Sample characteristics

The sample comprised 48 breast cancer survivors (mean age at diagnosis = 45.3 ± 10.9 years; Table 1). Racial/ethnic representation was evenly split between non-Hispanic White (41.7%, n = 20) and Black/African American women (41.7%, n = 20), with the remainder identifying as Hispanic/Latina (16.7%, n = 8). Most participants were highly educated: 77.1% (n = 37) held at least a college degree, including 41.7% (n = 20) with graduate or professional credentials. Slightly more than one-third were employed full-time at diagnosis (35.4%, n = 17), and 60.4% (n = 29) were married. Half of the participants reported an annual household income below $35,000, whereas one quarter earned more than $60,000. All participants had some form of health-insurance coverage. Clinical characteristics reflected predominantly early-stage disease: 85.4% (n = 41) were diagnosed with stage 0–II breast cancer. At the time of the survey, 54.2% (n = 26) were receiving active treatment. Nearly all had undergone surgery (83.3%, n = 40); 62.5% (n = 30) received chemotherapy and 41.7% (n = 20) radiation therapy. Time since diagnosis was ≤ 12 months for 62.5% of respondents, with 35.4% (n = 17) being more than one-year post-diagnosis.

**Table 1:** Sociodemographic and clinical/medical characteristics of the sample (*N*=48)

Participant Characteristics	Mean (SD)
Age at diagnosis	
Mean	45.30 (10.93
Race	% [*n*]
Black/African American	20 [41.67]
Hispanic/Latina	8 [16.67]
Non–Hispanic White	20 [41.67]
Education level	
Some high school	2.08 [1]
High school diploma	4.17 [2]
Some college/associate degree	16.67 [8]
College degree	35.42 [17]
Graduate/professional degree	41.67 [20]
Employment status	
Full–time	35.42 [17]
Others (e.g., work part time, on leave, other, etc.)	64.58 [31]
Marital status	
Married	60.42 [29]
Divorced	16.67 [8]
Single/never married/living with someone in marital–like relationship	22.91 [11]
Household income	
$10,000–$34,999	50.00 [24]
$35,000–$59,999	20.83 [10]
Over $60,000	25.00 [12]
No answer	4.17 [2]
Insurance coverage	
Yes	100.00 [48]
No	0.00 [0]
Treatment Status	
Active treatment	54.17 [26]
All others	45.83 [22]
Stage Status	
Stage 0	10.42 [5]
Stage I	41.67 [20]
Stage II	33.33 [16]
Stage III	12.50 [6]
Stage IV	2.08 [1]
Treatment	
Surgery	83.33 [40]
Chemotherapy	62.50 [30]
Radiation Therapy	41.67 [20]
Time since Diagnosis	
Less than 3 months ago	6.25 [3]
Between 3 and 6 months ago	12.50 [6]
Between 6 and 9 months ago	18.75 [9]
Between 9 and 12 months ago	25.00 [12]
More than 12 months ago	35.42 [17]
No answer/Missing	2.08 [1]

### Financial toxicity and correlates

**Fig. 1:**
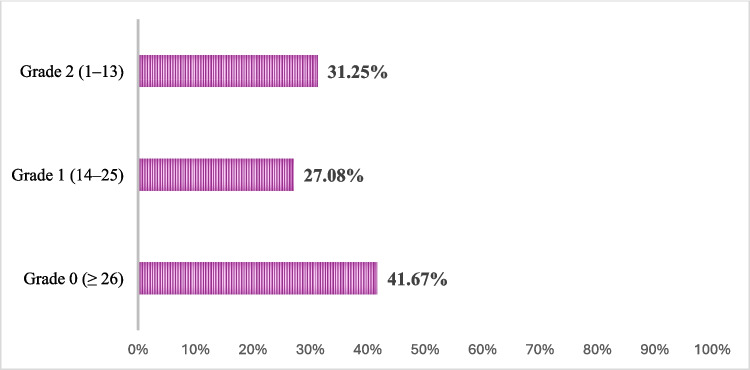
Distribution of Financial Toxicity Scores in the Study Sample. **Note:** No participant reported Grade 3 FT

Mean FT score was 21.77 ± 12.33 (possible range 0–44), and 58.33% reported Grades 1 and 2 FT (i.e., moderate to high levels; Fig. 1). Cronbach’s alphas ranged from 0.38 to 0.94 with almost all being greater than 0.7 except for three measures (emotion-focused coping, avoidant coping, and cancer care disruptions). Correlation analyses indicated that psychological distress (r = −0.450, *p* = 0.0013), emotion-focused coping behaviors (r = −0.331, *p* = 0.0215), avoidant coping behaviors (r = −0.299, *p* = 0.0392), self-efficacy (r = 0.337, *p* = 0.0192), HRQOL (r = 0.618, *p* < 0.0001), social isolation (r = −0.400, *p* = 0.0048), and cancer care disruptions (r = −0.540, *p* < 0.0001), were significantly correlated with FT. That is, women who reported greater psychological distress, and those experiencing more social isolation, having lower emotion-focused/avoidant coping behaviors, and greater disrupted cancer care delivery, respectively, reported severe financial toxicity. Women who reported greater self-efficacy and HRQOL reported a higher level of financial security (Table 2).

**Table 2:** Correlation analysis of key variables of interest

	Mean (SD)	Alpha	2	3	4	5	6	7	8	9
1. Financial Toxicity	21.77 (12.33)	0.91	–0.45**	–0.17	–0.33*	–0.30*	0.34*	0.62***	–0.40**	–0.54***
2. Psychological Distress	28.4 (6.99)	0.91	1	0.17	0.44**	0.51***	–0.51***	–0.65***	0.58***	0.27
3. Problem-focused Coping	3.12 (0.74)	0.89		1	0.59***	0.22	0.11	–0.11	0.06	0.11
4. Emotion-focused Coping	3.12 (0.74)	0.64			1	0.48***	–0.04	–0.36*	0.09	0.22
5. Avoidant Coping	2.7 (0.43)	0.61				1	–0.41**	–0.48***	0.17	0.28*
6. Self-Efficacy	84.42 (11.14)	0.73					1	0.61***	–0.55***	–0.28
7. Health-Related Quality of Life	112.85 (26.02)	0.94						1	–0.59***	–0.46**
8. Social Isolation	9.6 (3.98)	0.94							1	0.20
9. Cancer Care Disruptions	2.96 (0.73)	0.38								1

*SD* Standard Deviation; *p < 0.05; **p < 0.01; ***p < 0.001

### Regression analysis

Results of the final regression model (R^2^ = 0.69) showed that women who experienced greater HRQOL (β = 0.28, *p* < 0.001) were more likely to indicate financial stability. Compared with women with graduate degree or income ≥ $100,000, women with bachelor’s degree (β = −8.05, *p* = 0.01) or income < $50,000 (β = −6.92, *p* = 0.03) or $50,000-$99,999 (β = −8.07, *p* = 0.02) were more likely to report lower FT scores (i.e., less security of financial situation), respectively (Table 3).

**Table 3:** Backward selection linear regression analyses for financial toxicity and participants’ sociodemographic and psychosocial characteristics

	Estimate	SE	*t* value	p-value
Intercept	−2.32	6.41	−0.36	0.72
Race BAA (Ref: NHW)	2.05	2.74	0.75	0.46
Race HL (Ref: NHW)	4.52	3.63	1.25	0.22
Education Bachelor’s Degree (Ref: Graduate Degree)	−8.05	2.75	−2.92	**0.01**
Education High School and below (Ref: Graduate Degree)	−7.71	5.49	−1.40	0.17
Education Some College or Technical School (Ref: Graduate Degree)	−7.32	3.95	−1.85	**0.07**
Income $50,000—$99,999 (Ref: ≥ $100,000)	−8.07	3.26	−2.48	**0.02**
Income < $50,000 (Ref: ≥ $100,000)	−6.92	3.08	−2.24	**0.03**
Health-Related Quality of Life	0.28	0.05	5.79	** <.0001**

BAA = Black African American; HL = Hispanic Latino; NHW = Non-Hispanic White; SE = Standard Error

### Discussion

This study adds to the growing evidence that FT is both common and clinically relevant for breast cancer survivors. In the present study, almost two-thirds of women living with cancer reported moderate-to-high FT; a figure that sits above the 40-55% range documented in U.S. population-based cohorts [7, 8, 19] and the 28-48% prevalence observed when FT was evaluated by objective monetary indicators [50]. This discrepancy likely reflects both methodological and contextual factors. First, our sample was characterized by younger survivors and individuals with lower household income (50% reported household income below $35,000); subgroups that are consistently shown to shoulder a disproportionate economic burden [10, 11, 51]. Second, data collection overlapped in part with the early phase of the COVID-19 pandemic, a period characterized by job loss, medical care disruptions, and heightened out-of-pocket costs, factors associated with greater FT in prior studies, particularly among women and vulnerable groups [11, 26].

Observed correlations of financial toxicity mirrored prior reports from both high-income [52, 53] and low- and middle-income settings [54], with younger age, minoritized racial/ethnic identity, and lower income being over-represented among those reporting economic hardship [16, 17]. What diverged from the existing literature is the persistence of mild-to-moderate FT in a cohort with relatively broad insurance coverage and educational attainment, underscoring gaps in financial protection [55–57]. Additionally, no significant differences were detected among the three racial/ethnic groups in our sample, suggesting that self-reported FT may be influenced by complex interactions between individual, contextual, and societal factors. Collectively, these observations suggest that while our findings reinforce known risk gradients, they also highlight a substantial and concerning overall burden among breast cancer survivors navigating care in the pandemic aftermath.

Echoing this consideration, greater FT in our sample was closely associated with self-reported care delays. This pattern mirrors earlier works showing that out-of-pocket costs, high deductibles, and pandemic-related income losses prompt patients to postpone visits, forego imaging, or ration medications [15, 58, 59]; this is particularly concerning as even brief deferrals can jeopardize breast-cancer control and survival [60]. The pandemic intensified this dynamic: nearly 90% of oncology centers worldwide reported care disruptions, and almost half noted that > 10% of patients missed at least one treatment cycle [61]. An integrative review further documented that FT during COVID-19 precipitated difficulty covering basic expenses, heightened anxiety, and reduced income, while telemedicine sometimes offered a less burdensome alternative [62]. Breast cancer specific studies confirmed these trends, showing pronounced FT, especially among patients with metastatic disease, lower income, or greater depression severity [63]. These findings underscore how economic barriers and public-health crises converge to affect guideline-concordant care, regardless of insurance status. Proactive financial navigation, insurance-literacy coaching, and early referrals are therefore critical options for mitigating both FT and its downstream impact on treatment adherence [64–66].

Participants experiencing higher FT also endorsed significantly greater social isolation. Social networks often serve as informal safety nets; offering transportation, childcare, or short-term loans that can buffer financial strain [67–72]. When those ties are weakened or absent, survivors lose both emotional and practical resources, intensifying the economic burden of the illness [69, 73]. Empirical work further underscores the protective capacity of social and caregiver networks. Larger, more supportive circles are consistently associated with higher health-related quality of life and lower distress, in part because caregivers buffer the mental-health impact of cancer-related debt [67, 70, 74, 75]. Conversely, negative or absent caregiver responses predict poorer well-being [71, 72, 76], even years after diagnosis. Because FT itself is a strong determinant of diminished quality of life [55, 77], interventions that simultaneously strengthen survivors’ social networks and engage caregivers in coordinated financial counseling may deliver a dual benefit: reducing monetary hardship while reinforcing resilience [18, 78].

Finally, we observed that higher FT co-occurred with greater perceived stress and poorer HRQOL; a finding that is aligned with earlier reports [6, 57, 79–82]. Evidence suggests a bidirectional cycle in which economic hardship fuels psychological distress, while anxiety and depression reduce work capacity, erode income, and further amplify costs [83, 84]. Beyond its fiscal dimension, FT is consistently linked to clinically meaningful increases in anxiety, fatigue, and depression and to lower overall well-being among survivors [57, 85]. Breast cancer survivors, in particular, report more cognitive problems, sexual dysfunction, fatigue, and anxiety than women without cancer [86, 87]. Socioeconomic disadvantages and limited social support compound these effects, exacerbating mental-health burdens and diminishing quality-of-life outcomes [88, 89]. Accordingly, clinicians should adopt a biopsychosocial perspective: screening not only for financial strain but also for concurrent mental-health needs, social drivers of health, and functional limitations.

#### Limitations

Several limitations affect the interpretation of our findings. First, the cross-sectional design cannot establish temporality or causality between FT, care delays, and psychosocial factors. Therefore, we are unable to determine whether the observed burden represents a shift attributable to the pandemic. Rather, our findings should be interpreted as characterizing the experience of breast cancer survivors navigating the pandemic context. Second, the modest sample size limited statistical power for nuanced subgroup analyses, raising the possibility that some associations went undetected. Third, these recruitment strategies generated a convenience sample that, despite racial/ethnic diversity, may not represent rural residents, uninsured patients, or non-English speakers, thereby constraining generalizability. The relatively high educational attainment and universal insurance coverage in this sample may underestimate the magnitude of FT experienced by individuals with lower socioeconomic status, who have been disproportionately affected by both cancer-related costs and pandemic-related economic instability. Fourth, reliance on self-reported questionnaires introduces recall and social-desirability bias, and the absence of objective financial indicators (e.g., billing records, credit reports) may underestimate the full economic burden. Future longitudinal, multi-site studies with larger samples are needed to map trajectories of FT through long-term survivorship and to test variations across racial, ethnic, linguistic and cultural groups. Incorporating objective financial metrics alongside patient-reported outcomes will strengthen construct validity and enable cost-effectiveness modelling. Studies should also evaluate how financial navigation services, policy interventions, and community-based programs can alleviate financial concerns. Finally, qualitative work could illuminate contextual factors that quantitative surveys may miss, providing a richer foundation for program development.

## Conclusion

The present work documents that for women diagnosed with breast cancer, the experience of navigating treatments and health care services during the pandemic was characterized by considerable financial distress. Healthcare system characteristics, lower income and education, next to psychosocial factors were associated with self-reported financial burden. While these results should be considered in the context of extant study limitations, they also provide further confirmation for the need for health care teams to address the complex cost of oncology care, especially at a time of heightened financial uncertainty and erosion of Medicare and Medicaid capacity [90, 91]. A multidisciplinary, person-centered approach to financial toxicity management can extend current navigation models by addressing some of the psychosocial and behavioral factors highlighted in this analysis. At the same time, providers must be supported to screen for and manage financial hardship across the continuum of care. Institutions can reinforce these efforts through investment in financial navigation services; professional societies should integrate value and affordability considerations into trial design and guideline development; and policymakers have a critical role in advancing reforms that improve affordability, expand insurance protections, and safeguard patients and family caregivers.

## Data Availability

The de-identified dataset that supports the findings of this study is available from the corresponding author on reasonable request.
